# Biodegradable CSMA/PECA/Graphene Porous Hybrid Scaffold for Cartilage Tissue Engineering

**DOI:** 10.1038/srep09879

**Published:** 2015-05-11

**Authors:** JinFeng Liao, Ying Qu, BingYang Chu, XiaoNing Zhang, ZhiYong Qian

**Affiliations:** 1 State Key Laboratory of Biotherapy and Cancer Center, West China Hospital, Sichuan University, and Collaborative Innovation Center for Biotherapy, Chengdu, 610041, China; 2 School of Medicine, Tsinghua University, Beijing, 100084, China

## Abstract

Owing to the limited repair capacity of articular cartilage, it is essential to develop tissue-engineered cartilage for patients suffering from joint disease and trauma. Herein, we prepared a novel hybrid scaffold composed of methacrylated chondroitin sulfate (CSMA), poly(ethylene glycol) methyl ether-ε-caprolactone-acryloyl chloride (MPEG-PCL-AC, PECA was used as abbreviation for MPEG-PCL-AC) and graphene oxide (GO) and evaluated its potential application in cartilage tissue engineering. To mimic the natural extracellular matrix (ECM) of cartilage, the scaffold had an adequate pore size, porosity, swelling ability, compression modulus and conductivity. Cartilage cells contacted with the scaffold remained viable and showed growth potential. Furthermore, CSMA/PECA/GO scaffold was biocompatible and had a favorable degradation rate. In the cartilage tissue repair of rabbit, Micro-CT and histology observation showed the group of CSMA/PECA/GO scaffold with cellular supplementation had better chondrocyte morphology, integration, continuous subchondral bone, and much thicker newly formed cartilage compared with scaffold group and control group. Our results show that the CSMA/PECA/GO hybrid porous scaffold can be applied in articular cartilage tissue engineering and may have great potential to in other types of tissue engineering applications.

The poor regenerative and reparative ability of articular cartilage following injury or degenerative diseases makes this tissue a key target for cell-based therapy and tissue engineering^1^,^2^. Articular cartilage has an avascular structure that limits its self-healing capacities once damaged. Current strategies of articular cartilage repair in clinical situation include the use of expanded autologous chondrocytes injected to the lesion underneath a periosteal flap, or attached to a scaffold support^3^. These clinical options are often inadequate long-term, and thus, alternative treatment based on tissue engineering strategies may significantly improve patient care. Consequently, strategies based on exogenous scaffold, a critical element in tissue engineering, have been investigated for cartilage repair^4^. Scaffolds play a role similar to the extracellular matrix (ECM) in natural tissues, supporting cell attachment, proliferation and differentiation. The architectural design of scaffolds may create an environment that can preserve the normal phenotype of cells to promote regeneration of cartilage-like constructs^5^.

Nowadays, many natural^6^,^7^,^8^,^9^ and synthetic^10^,^11^,^12^ polymers are widely applied to prepare scaffolds. Naturally derived polymer chondroitin sulfate (CS) is the component of naturally occuring cartilage, and ECM is known to influence the proliferation and differentiation of chondrocytes^13^,^14^. CS plays an important role in regulating the expression of the chondrocytes phenotype and is also involved in intracellular signaling, cell recognition and the connection of ECM components to cell-surface glycoproteins, which can be useful for guiding tissue repair and regenerative medicine applications^15^. The previous study^16^ showed that gelatin-chondroitin-hyaluronate copolymer, which mimicked the normal ECM of cartilage, could enhance cellular adhesion, chondrogenic differentiation and glycosaminoglycan (GAG) synthesis. Besides, CS can be used as a novel bioadhesive to the tissue protein^17^. Methacrylate and aldehyde groups were functionalized on the polysaccharide backbone of CS to chemically bridge biomaterials and tissue proteins via a twofold covalent link.

Regarding polymeric materials, poly(ε-caprolactone) (PCL) has been widely investigated in conventional form to repair the full-thickness cartilage defect and showed fairly good results in vivo. Chondrocytes also showed proliferation and ECM synthesis on PCL scaffold in vitro culture^18^,^19^. In addition, PCL is one of a few synthetic materials that have been used in products approved by the Food and Drug Administration (FDA) for clinical applications. PCL is highly appealing due to its unique physico-chemical and mechanical characteristics^20^. It has been previously reported that chondrocytes attached successfully and proliferated on PCL films^18^ and, additionally, started to produce a cartilaginous ECM in PCL scaffolds^21^. Also, another strategy allowed the fabrication of a combination cell-therapy implant capable of robust and durable cartilage repair in large defects^22^. The electrospun PCL scaffold was enrobed with nanoreservoirs to entrap, protect, and stabilize bone morphogenetic protein (BMP-2). Upon contact with cells, BMP-2 became available through enzymatic degradation of these nanoreservoirs. However, the main drawback of PCL as a scaffold material is the absence of cell recognition sites, considering its hydrophobicity and relatively slower degradation/resorption kinetics compared to other polyesters^23^. In previous work, our group have successfully prepared methoxyl poly(ethylene glycol)-poly(ε-caprolactone)-acryloyl chloride (MPEG-PCL-AC, PECA was used as abbreviation for MPEG-PCL-AC) amphiphilic macromonomer and integrated it the main constituent of a hydrogel^24^. By incorporating PEG segment into PECA copolymer backbone, the hydrophobicity and biodegradation rate of the hydrogel was improved^25^.

To date, scaffolds do not govern their biocompatibility, degradation, mechanical function and bioinductive properties^26^ all at the same time. They are usually composed or only synthetic materials or natural polymers, while synthetic materials can provide biomimetic mechanical properties and predictable degradation profiles, they generally possess a limited capacity to interact with cells without modification or functionalization with defined ligands^27^. On the other hand, native extracellular matrices present an attractive biomaterial option for scaffold fabrication because they offer a host of tissue-appropriate physical and chemical cues that govern specific cell proliferation, differentiation, and matrix synthesis^28^. To explore a more appropriate scaffold, we designed a scaffold, which consists of methacrylated chondroitin sulfate (CSMA) and PECA, along with graphene oxide (GO), as shown in Fig. 1. GO acts as a supplementary material in our scaffold as the emerging carbon nanomaterials offer numerous opportunities to design novel scaffolds for tissue engineering^29^,^30^,^31^,^32^,^33^. GO is known for its high-mechanical strength, large surface area, electrical property and its ease for chemical modifications^34^,^35^,^36^. The graphene aerogel obtained with liquid nitrogen freezing retained its fine, cellular structure, even after 10 cycles of compression under 1 N weight (~7 KPa pressure)^30^. In the field of cartilage tissue engineering, GO could be one promising candidate for novel scaffold as it may enhance the topographical, mechanical and electrical cues in the scaffold to provide an environment for tissue regeneration that is superior to conventional inert biomaterials. Wang and his coworkers^37^ incorporated GO into their composite, which produced a self-healing nanocomposite with good mechanical strength. They found that the advantage of using GO instead of other typical crosslinkers is that only a small amount of GO was needed to achieve a dramatic improvement in the overall mechanical property of the composite, due to the multiple reactive sites on GO and its high mechanical strength. Also, Nayak *et al.*^38^ reported that graphene provided a promising biocompatible scaffold that did not hamper the proliferation of human mesenchymal stem cells (hMSCs) and accelerated their specific differentiation into bone cells. In this regard, GO holds great potential and was selected as a supplementary material for our hybrid scaffolds.

The CSMA/PECA/GO scaffold has macro-pore dimensions with highly interconnective morphology to mimic the three-dimensional environment of the cartilage. The hybrid scaffold also had good mechanical property and favorable degradation rate that match the rate of healing of the damaged tissue. The scaffold was biocompatible with good adhesion and allowed the proliferation of cartilage cells in vitro. Futhermore, the scaffold was implanted into full-thickness cartilage defect in a rabbit model to evaluate the regeneration of defect in vivo, as displayed in Fig. 2.

## Results and Discussion

### Characterizations of CSMA/PECA/GO hybrid scaffolds

**Figure 3:**
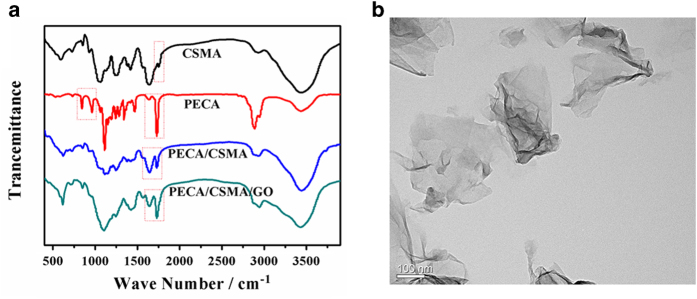
(**A**) FTIR of CSMA, PECA, CSMA/PECA and CSMA/PECA/GO hybrid scaffold. (**B**) TEM image of GO.

The PECA copolymer was synthesized in a similar way to our previous work^39^, which proved to be a biocompatible material with low cytotoxicity. CSMA was formed by using glycidyl methacrylate to conjugate with CS. The CSMA/PECA/GO scaffold was prepared by the heat-initiated free radical method with APS as the initiator agent. Then it was characterized by IR spectroscopy and, the FTIR spectra of the components and scaffold, as shown in Fig. 3A. CS, is distinguished by saccharide alkyls at ~2,920 and ~1,416 cm^−1^, and alkoxyls at ~1,030 cm^−1^. When the CS was modified with the functional groups, a new infrared band associated with the C = O stretch appears at ~1,736 cm^−1^. The absorption peaks at ~1,746 cm^−1^ and ~1,122 cm^−1^ are respectively assigned to ester stretching vibrations of PECA. The absorption signals at ~1,621 cm^−1^ and ~850 cm^−1^ are attributed to C = C stretching vibration of PECA. However, the C = C stretch weakened obviously in the spectrum of CSMA/PECA scaffold and the result indicated that most of the carbon-carbon double bonds in PECA had cross-linked and converted into carbon-carbon single bonds which formed the main chain of scaffold without GO. On the other hand, the C = C vibration of CSMA/PECA/GO scaffold became stronger, due to the skeletal vibration of GO. Also, a typical micrograph (TEM) of the GO used in this engineered scaffold is shown in Fig. 3B.

**Figure 4:**
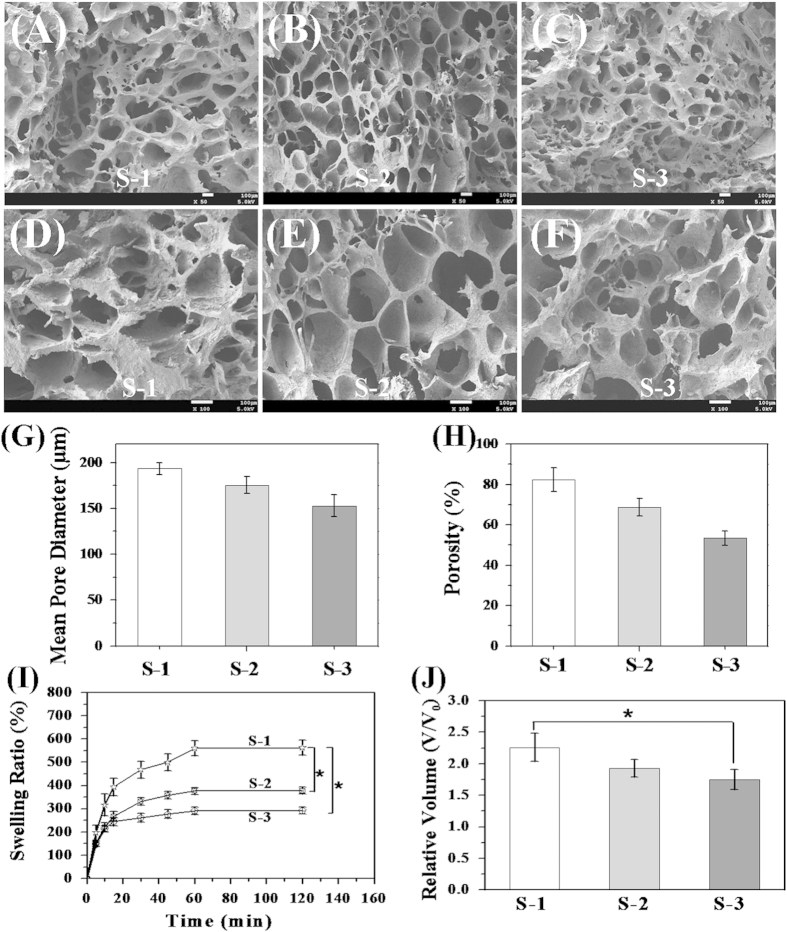
(**A**–**F**) SEM micrographs of the cross section of virious hybrid scaffolds (S-1, S-2, S-3). Scale bars are 100 μm. (**G**) pore diameters, (**H**) porosities, (**I**) swelling abilities and (**J**) volume changes of different amount monomers in CSMA/PECA/GO scaffolds.(*p < 0.05).

An ideal scaffold should be able to mimic the ECM of cartilage, which was important to keep the phenotype of differentiated MSCs or cartilage cells. The optimum scaffold should meet certain criteria, such as provide a suitable 3D structure for cell growth and nutrient transport^40^,^41^,^42^. Following the same rationale, porous CSMA/PECA/GO scaffold with different proportion of monomers (Table 1) were fabricated S-1, S-2 and S-3. A highly porous and interconnected network structure formed by lattice-like holes was observed by SEM (Fig. 4A-F). From S-1 to S-3 (Fig. 4G), the mean pore sizes were 193.5 μm, 175.2 μm and 152.8 μm, respectively. The pores were fairly uniform and round-shaped. The pore size of bioscaffold is suitable to seeding capability, as current design/fabrication occurs at scales above 100 μm^43^. The pore sizes decreased with the decreasing proportion of CSMA and PECA. The pore size of the scaffolds mainly depends on the water content of the initial CSMA and PECA blending solution, especially due to CSMA (the hydrophilic ability of CSMA). The porosity was high as measured by mercury intrusion porosimetry. The mean porosity of scaffolds increased as the CSMA content increased (Fig. 4H).

Figures 4I,J show the swelling ability and volume changes properties of different amount of CSMA and PECA in CSMA/PECA/GO scaffold. The swelling ratio and volume change relate to the reabsorption of water content. The samples were lyophilized before the test. The scaffolds with varying CSMA and PECA all reached swelling equilibrium within 1 h. With the increase of CSMA, the scaffolds showed higher swelling and volume. This can be due to the hydrophilic ability of CSMA. The higher water adsorption capacity, can prevent the loss of body fluid and nutrients when the scaffold is applied in the defect repair in vivo. The variations in the water adsorption of the synthesised scaffold were similar to those of the porosity. It might be explained that the CSMA/PECA/GO scaffold made from high CSMA concentration had higher porosity and larger pore size, which offered more space for water storage, yielding an increase in water adsorption. Although the swelling ratio and volume change are expected to resemble the in vivo abilities. When filling a defect, the scaffold will swell under confined conditions, in which case swelling is controlled by the stiffness of the surrounding tissue. Generally very little swelling was observed in an articular cartilage defect^15^.

**Figure 5:**
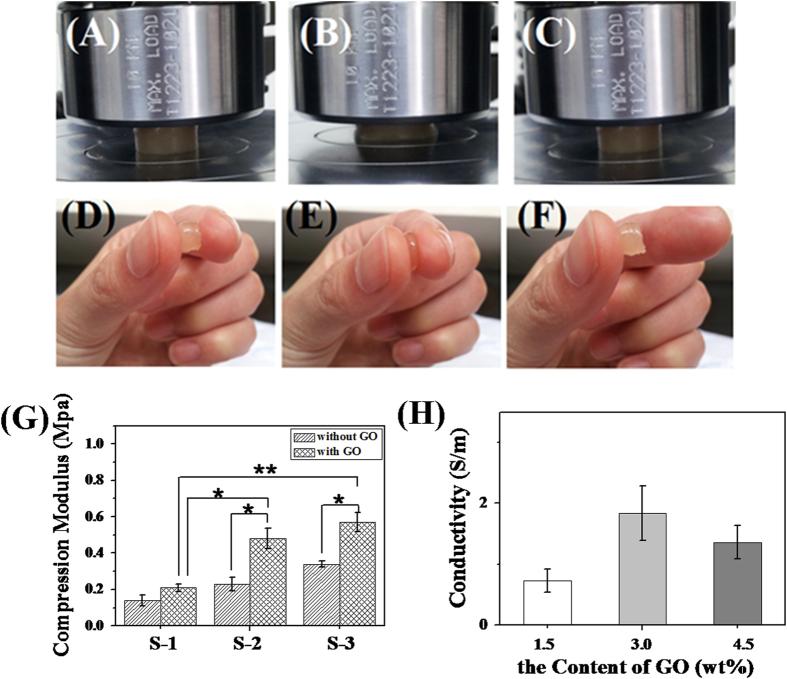
Images of the CSMA/PECA/GO scaffold sample (S-2) (**A**,**D**) before unconfined compression, (**B**,**E**) at 65% strain and (**C**,**F**) immediately after compression. (**G**) compression modulus (GO 3.0 wt%) and (**H**) electrical conductivity of different amount GO in S-2 scaffold.(*p < 0.05, ** p < 0.01).

To serve its function as a biomechanical structure articular cartilage is exposed to a variety of forces such as hydrostatic pressure, compression and shear forces^44^,^45^,^46^. Based on these facts, the biomechanical properties of engineered constructs was examined by unconfined dynamic compression. The deposition of matrix within constructs is related to the development of frequency-dependent dynamic compressive behavior indicative of viscoelastic properties that are important for the proper biomechanical function of articular cartilage^47^. Samples from one formulation (S-2) had a compressive modulus of 0.48 MPa and did not fracture at all with 65% compression (Fig. 5A–F). Immediately after compression, the sample recovered to approximately 98% of its original height. S-1 and S-3 had a compressive, load of 54% and 72% before they fractured. The compressive moduli of the different scaffolds are displayed in Fig. 5G. The compressive strength of scaffold increased from S-1 to S-3 with the increase of PECA content, which might be attributed to the mechanical characteristic of PECA. For human motion, the average loading on knee and hip joints for normal movement is 0.5-7.7 MPa^48^. The modulus of scaffolds (S-2 and S-3) can almost satisfy this demand. Meanwhile, the samples can stick to the skin indicating that the scaffold may act as a glue for cartilage tissue engineering (Fig. 5F), which may attributed the remaining methacrylate and aldehyde groups on the polysaccharide backbone of CS to chemically bridge the scaffold and tissue proteins^17^. On the other hand, GO also play a critical role in the modulus of scaffold. With 3.0% GO in scaffolds, the compressive modulus almost doubled compared with scaffolds without GO (for S-2 and S-3). Moreover, adding GO into the scaffolds can improve the electrical conductivity of the scaffolds (Fig. 5H). The conductivity of the scaffolds without GO was undetectable. While the electrical conductivity of the CSMA/PECA/GO scaffolds with 1.5% GO was ~0.73 S/m. The increase of GO did also increase the conductivity of the scaffolds. When the content of GO was 3.0% in the scaffold, the conductivity was ~1.84 S/m. But flocculent precipitates were found in the scaffold as GO was further increased to 4.5%, which resulted in a decrease of the conductivity. Considering these results, we chose 3.0% of GO to add into the scaffolds for future experimentation. Also, on the comprehensive evaluation of all the results obtained, S-2 was selected for further study which had relatively high pore size, porosity, appropriate swelling ability and volume change property, good compressive ability and electrical conductivity.

### Cell cytotoxicity and biodegradablity of CSMA/PECA/GO hybrid scaffold

**Figure 6:**
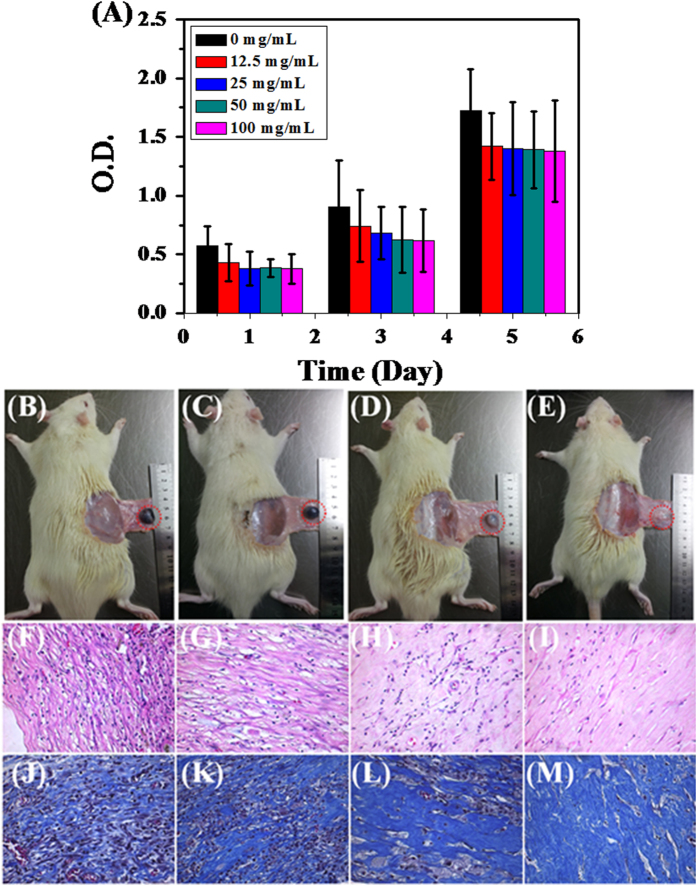
(**A**) Cell viability of 3T3 cells in the leachates of scaffold (0, 12.5, 25, 50, 100 mg/mL) at 1, 3 and 5 days. The photographs of subcutaneous implanted CSMA/PECA/GO scaffold on the back of mice for (**B**) 1, (**C**) 2, (**D**) 4 and (**E**) 8 weeks. Histological section of subcutaneous implanted CSMA/PECA/GO scaffold for different periods. Images **F**, **G**, **H** and **I** were of H&E staining at 1, 2, 4 and 8 weeks. (**J**), (**K**), (**L**) and (**M**) were of Masson staining at the same time as H&E staining (Original magnification × 400).

Since cartilage substitutes commonly serve as a temporary replacement for the ECM, they should have excellent biocompatibility and suitable biodegradability. Cell viability of 3T3 cells in presence of the leachates of hybrid scaffold for 1, 3 and 5 days was analyzed using MTT assay (Fig. 6A). Although cell viabilty cultured in the leachates of scaffold had a percentage about 80% when compared with control (0 mg/mL), viability and metabolic activity of 3T3 cells cultured in the different concentration of extractions showed a significant increase on 1 day and 3 days after seeding. The cells remained proliferated slightly up to day 5. The high viability indicates that the prepared scaffold was biocompatible. To investigate in vivo degradability and biocompatibility, CSMA/PECA/GO scaffolds were implanted subcutaneously into a rat animal model. As displayed in Fig. 6B–E, the morphology of scaffold changed progressively with time after implantation. At 1 week and 2 weeks, part of the implanted composite was absorbed. Along with the implantation, the degradation of hybrid scaffold happened as expected. Two months were needed for the scaffold to completely degrade. The degree of inflammation elicited by the engineered scaffold material was evaluated by H&E staining (Fig. 6F–I). Some inflammatory cells could be seen in the material at 1 week, revealing that an acute inflammation response (leukocyte infiltration) occurred. The inflammation response had reduced after 2 weeks. And no obvious inflammatory reaction could be seen after embedding for 4 weeks. No any signs of hematoma and purulent activity was found in whole process of implantation and leukocyte cells disappeared after 4 weeks, revealing that the composite is relatively safe to surrounding tissues. Figure 6J–M were the Masson staining at the same time points as H&E, which acted as a supplemental investigation to reveal the biocompatibility of hybrid scaffold. The blue-dyed collagen was uniformly distributed into the composite and it gradually expanded with the degradation of the scaffold. At 8 weeks post-implantation (Fig. 6M), the collagen has spread out equably as a part of newly fibrous tissues.

### Cell culture on CSMA/PECA/GO hybrid scaffold

**Figure 7:**
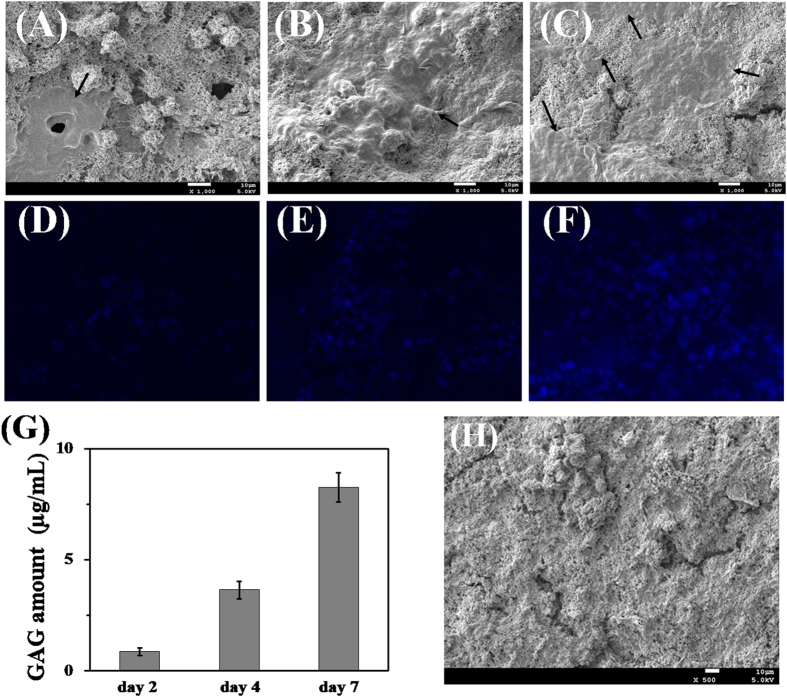
(**A**, **B** and **C**) SEM photographs and (**D**, **E** and **F**) the fluorescence images of chondrocytes incubation on the surface of CSMA/PECA/GO scaffold after culturing for 2, 4 and 7 days, (The arrows show chondrocytes). (**G**) GAG quantification assays after 2, 4 and 7 days of culture. (**H**) SEM photograph of the surface of CSMA/PECA/GO scaffold.

The cell proliferation proile of the scaffold was investigated as it is critical for a scaffold to support and guide the regeneration of tissues. SEM observation of the cartilage cells-seeded scaffold (Fig. 7A), in which the cartilage cells were cultured for 2 days, revealed that the cells well-adhered to the surface of the scaffold. The roughness of the scaffold surface (Fig. 7H) was attributed to GO and the natural component CS and likely played a role in cell attachment. On the day following cell seeding event, the cartilage cells already expanded onto the surface of the hybrid scaffolds (Fig. 7B,C). Subsequently, it became extremely difficult to line out the exact boundary of cells since the cells grew adherent massively to each other and formed a continuous layer. The fluorescent microscopic images of nucleus stained cells (Fig. 7D–F) also clearly indicate a significant increase in cell number. On the other hand, the synthesis of GAGs, is an important function of chondrocytes and plays a significant role in regulating the chondrocyte phenotype. Figure 7G shows that the GAG content increased significantly for the culturing times used. It suggests that the hybrid scaffold is compatible and provides a microenvironment that enables chondrocytes to anchor and proliferate^11^.

### Full-thickness cartilage defect repair

**Figure 8:**
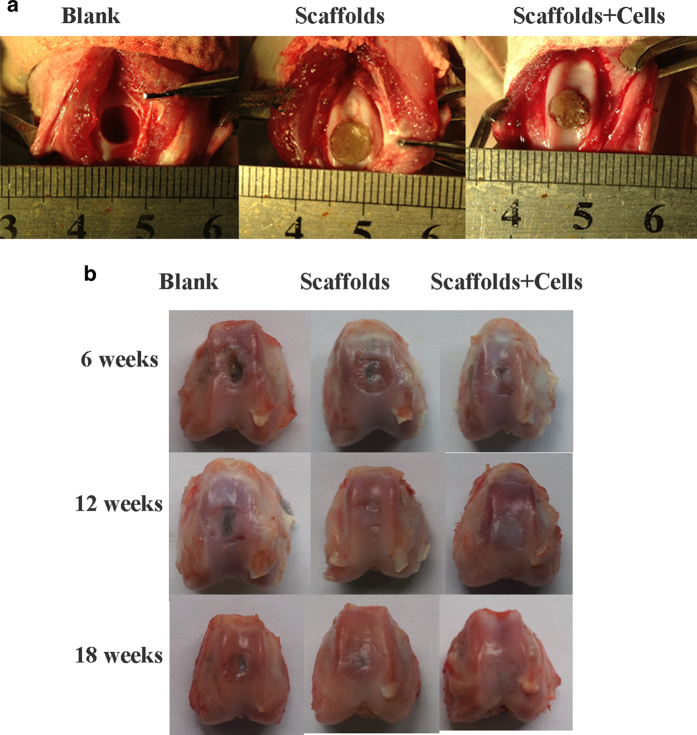
Photographs of the knee joint in different groups (**A**) at operation and (**B**) post-operation for 6, 12 and 18 weeks.

Finally, a rabbit model was used to evaluate the potential of CSMA/PECA/GO scaffolds in a critical-sized osteochondral defect (Fig. 8A). The cartilage regenerative capability was assessed by means of gross morphology examination, Micro-CT reconstruction evaluation and semi-quantitative histological scoring analysis, as well as histological immunohistochemical evaluation.

Macroscopically, the defects of blank group appeared depressed and irregular with poor integration with the host cartilage by 6 weeks, the scaffolds + cells treated groups showed the defects were both filled with white transparent tissue, but were not fully filled up (Fig. 8B). After 12 weeks the defects repair, the scaffolds + cells group appeared with tissue graft of moderately flat surface regularity. In contrast, the control group with empty defect displayed incomplete tissue coverage, while scaffolds treated group displayed moderate tissue coverage. At 18 weeks postoperatively, the scaffolds + cells treated defect filled with white translucent cartilage tissue which appeared smooth and well embedded with the surrounding normal cartilage. In contrast, the control groups displayed complete tissue coverage or treated with scaffolds showed irregular articular surfaces, whose integration of the newly formed cartilage plug with host cartilage was not very well accomplished.

**Figure 9:**
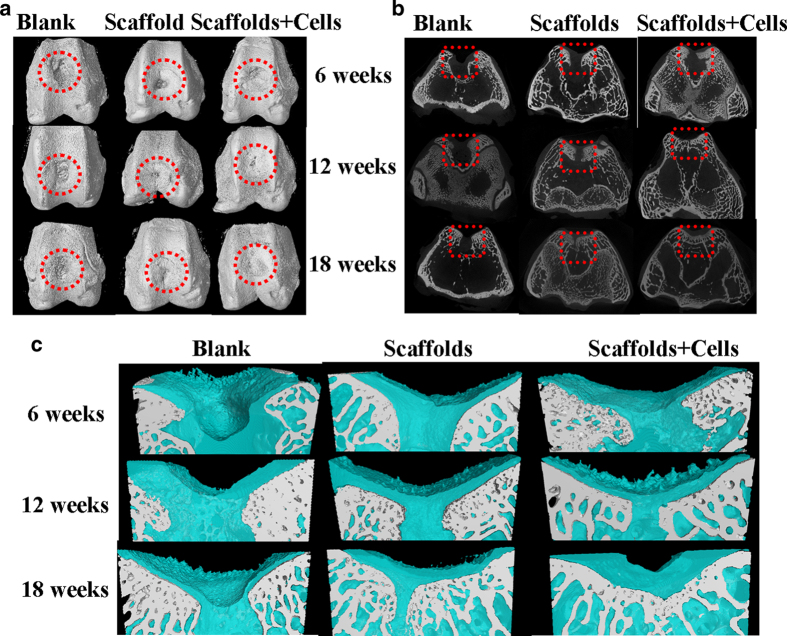
Micro-CT obtained from (**A**) 3D reconstruction and (**B**) 2D reconstruction in cross section of the articular joint (red circles and red squares refer to the part of full-cartilage defect) and (**C**) 3D reconstruction of the repaired full-cartilage defect (Cartilage and bone growth are denoted blue and grey, respectively.) at post-operation for 6, 12 and 18 weeks.

**Figure 10:**
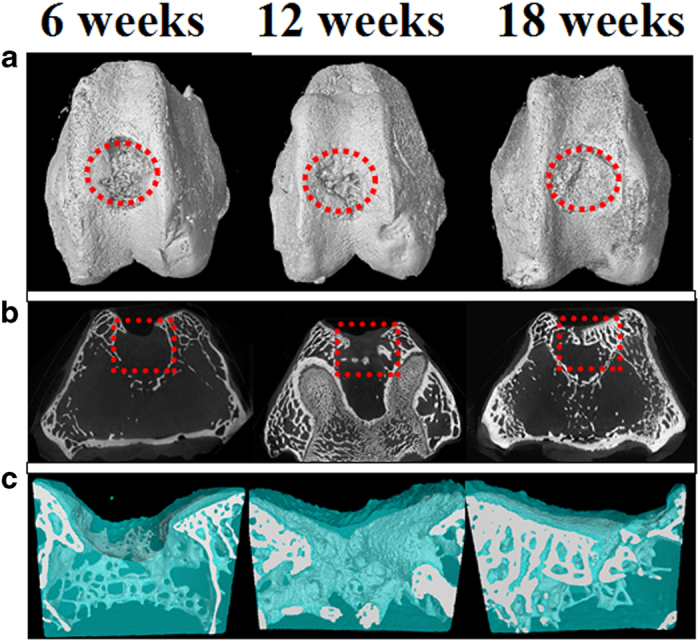
Micro-CT images of cartilage cell supplement group obtained from (**A**) 3D reconstruction and (**B**) 2D reconstruction in cross section of the articular joint (red circles and red squares refer to the part of full-cartilage defect) and (**C**) 3D reconstruction of the repaired full-thickness cartilage defect in cell supplement group (Cartilage and bone growth are denoted blue and grey, respectively.) at post-operation for 6, 12 and 18 weeks.

Micro-CT reconstruction was performed for the articular joint samples at each time point as shown in Fig. 9. In our study, the used defect model was a full-thickness osteochondral defect, which extend deep beneath the tidemark, but did not penetrate the subchondral bone plate in the bone marrow^49^. Therefore, the cartilage and part of the subchondral bone were the defects in this model. From the Micro-CT images of the 3D and 2D reconstruction of the knee joint (Fig. 9A,B), the newly bone mainly regenerated from the edge of defects towards the center. 18 weeks after surgery, the defect treated with scaffolds + cells was basically filled with newly cartilage and bone tissue, in comparison, a depression zone still maintained in the control group. The 3D reconstruction of the cartilage defects were isolated out in Micro-CT models (Fig. 9C). The growth of cartilage and subchondral bone can be clearly distinguished by the denoted colors. In the early period of post-operation, the cartilage formed firstly and the scaffold degraded gradually. Then the cartilage turned to subchondral bone after calcification. In the scaffold + cells repaired group, the defect was fully repaired and grew a new layer of cartilage. The cell group was also investigated, as shown in Fig. 10. The defect always presented as a depression hole even after implantation of the cartilage cells at 6 and 12 weeks. There was no significantly difference between the blank control group and cell group.

**Figure 11:**
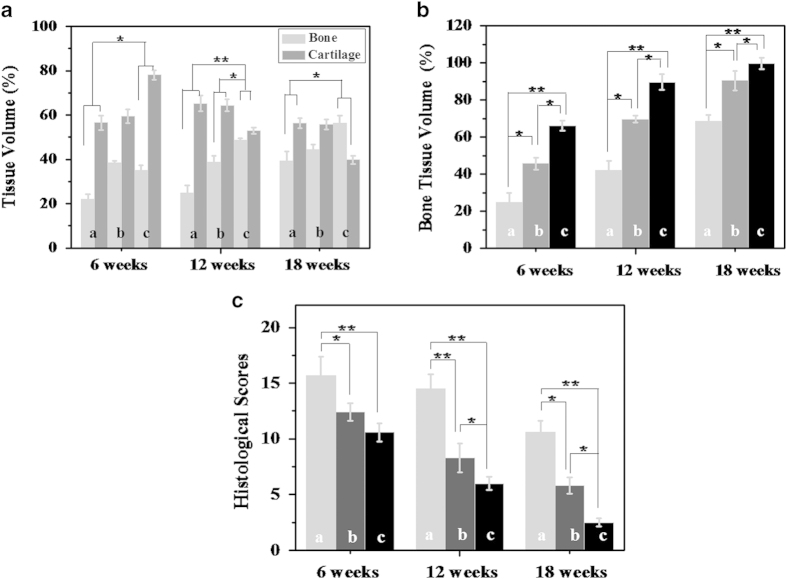
(**A**) The cartilage and bone portions of the reparative tissues were segmented using the thresholding function, and their respective 3D volumes were computed from the segmented region of interest (ROI); (**B**) Semi-quantitative analysis the relative amount of bone and cartilage tissues formation in the critical size cartilage defect model based on Micro-CT images; (**C**) Histological scoring for reparative tissues. The reparative tissues were evaluated for cell morphology, matrix-staining, surface regularity, subchondral bone reconstruction, filling of defect and integration of donor with host adjacent cartilage. (a, b and c indicate blank group, scaffolds group and scaffolds + cells group. *p < 0.05, ** p < 0.01).

The quantitative data about cartilage and subchondral bone proportion and volume also could be computed from the segmented region of interest (ROI). Characteristic remodeling processes marked by changes in the ratio of cartilage to subchondral bone volume of the repaired tissue and the percentage of bone tissues (including cartilage and subchondral bone) was observed. The efficacy of cartilage repair is one of the most important characteristic of bone substitutes. The percentage of cartilage and subchondral bone volume of the scaffolds + cells treated defect progressed from 78.0% cartilage and 35.1% subchondral bone at 6 weeks post-implantation to 53.0% cartilage and 48.6% subchondral bone at 12 weeks post-implantation, (Fig. 11A). And the total volume of bone tissue in this group occupied the most area of the defect compared with the scaffolds treated group and blank group (Fig. 11B). Moreover, the scaffolds + cells regenerated osteochondral tissue at 18 weeks resembled closely to the age-matched unoperated native articular tissue (cartilage 40.2% and subchondral bone 59.8%)^50^ of 39.8% cartilage and 56.3% subchondral bone.

The histological score data further demonstrated that the scaffolds containing cartilage cells did improve the repair capacity of full-thickness defects. The histological scores were evaluated for cell morphology, matrix-staining, surface regularity, subchondral bone reconstruction, filling of defect and integration of donor with host adjacent cartilage. In comparison with the empty control and scaffolds group, the scores of scaffolds + cells implantation group were better (i.e. lower) at 6, 12 and 18 weeks post-operation (Fig. 11C). At 6 weeks, the overall histological scores for blank group, scaffolds group and scaffolds + cells group were 15.7, 12.4 and 10.1, respectively. By 12 weeks, the overall histological scores scaffolds group and scaffolds + cells group were significantly better than those of control. Furthermore, there was significant improvement in score of scaffolds + cells group at 18 weeks when compared to blank or scaffolds group, indicating that the engineered cartilage underwent a dynamic remodeling process over 18 weeks, enhancing its reparative effect.

**Figure 12:**
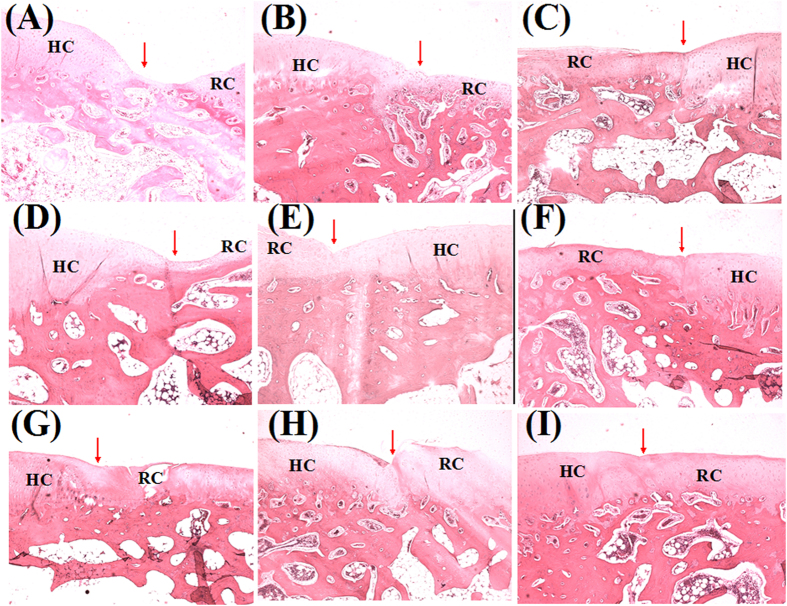
H&E staining of cartilage repair in (**A**, **D**, **G**) blank group, (**B**, **E**, **H**) scaffolds group and (**C**, **F**, **I**) scaffolds + cells group at postoperative (**A**, **B**, **C**) 6 weeks, (**D**, **E**, **F**) 12 weeks, and (**G**, **H**, **I**) 18 weeks. (Original magnification × 100; RC: repaired cartilage, HC: host cartilage.) Note: the arrows point to the repair interface.

**Figure 13:**
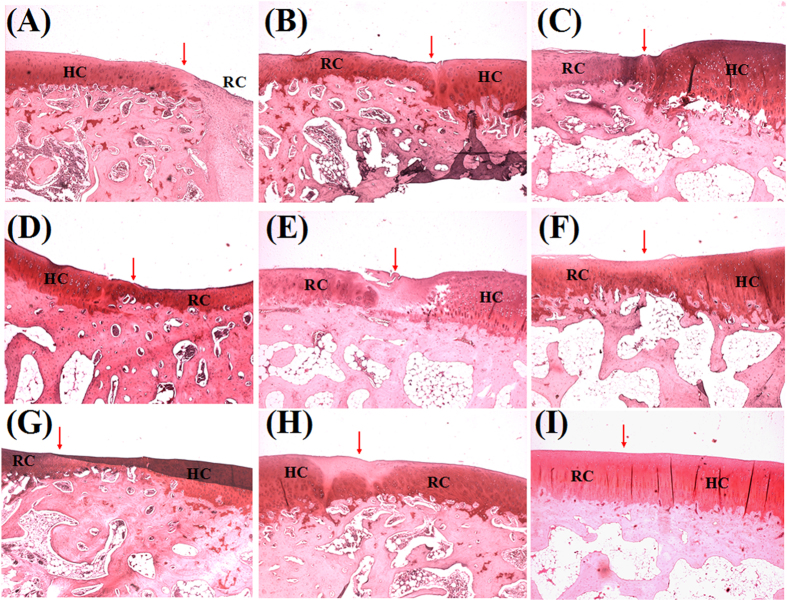
Safranin-O staining of cartilage repair in (**A**, **D**, **G**) blank group, (**B**, **E**, **H**) scaffolds group and (**C**, **F**, **I**) scaffolds + cells group at postoperative (**A**, **B**, **C**) 6 weeks, (**D**, **E**, **F**) 12 weeks, and (**G**, **H**, **I**) 18 weeks. (Original magnification × 100; RC: repaired cartilage, HC: host cartilage.) Note: the arrows point to the repair interface.

Histologically, 6 weeks after transplantation of the scaffolds + cells, chondrocytes distributed uniformly but aligned disorderly in defects, but almost no type growth in the empty defects by H&E staining (Fig. 12). Some chondrocytic cells could be observed in the scaffolds-treated defects as observed. At 12 weeks, the scaffolds + cells treated group regenerated a smooth cartilage surface with comparable matrix staining to the adjacent host cartilage. These repaired tissues exhibited uniform staining for GAG by Safranin-O staining (Fig. 13), when compared to the results at 6 weeks. The scaffolds group also showed recovered subchondral bone and hyaline cartilage, it was not worth that the new cartilage was thinner than the one of scaffolds + cells group. At 18 weeks post-operation, the chondrocytes lost their typical morphology and appeared some fibrocartilage-like cells in upper layer of newly formed cartilage in the control group. The scaffolds + cells group demonstrated the thickness of cartilage comparable to the normal one and matrix was extensively metachromatic. In addition, the subchondral bone and morphology of chondrocytes were even normal. The boundary of the host cartilage and the repaired cartilage can not be distinguished by the uniform staining of GAG (The host cartilage had the uniform staining of GAG and smooth cartilage surface. The repaired cartilage may not as well as host cartilage, such as the nonuniform staining of GAG, the thinner of cartilage, the roughness surface of cartilage, or depressed subchondral bone regeneration.). There was almost complete subchondral bone regeneration, marked by new bone formation sequestered within the matrix network of hypertrophic cartilage. The repair of scaffolds group had relative thinner newly formed cartilage and even more irregular surface. Meanwhile, the connection of the repaired cartilage and host cartilage was not integrity. As the reconstitution of hyaline cartilage and superficial zone were a prerequisite for the prolonged integrity of the repaired cartilage, it is possible that hybrid scaffold helped to maintain cell growth activity and prevent degeneration^51^. We speculated the group of scaffold + cells could recruit more MSCs and maintain chondrocytes characteristics. Meanwhile, the use of chondrocytes could enable immediate synthesis of matrix in the defects.

## Conclusion

In conclusion, this study fabricated a novel CSMA/PECA/GO hybrid scaffold, which mimicked ECM to serve as scaffold for cartilage tissue engineering. This scaffold manufactured with high porosity, elasticity, swelling ratio, electrical conductivity and rational degradable time, which creates a suitable environment for the regeneration of tissue-engineered cartilage. Cartilage cells seeded on the hybrid scaffold retain chondrogenic property, which in turn led to a better remodeling of regenerated cartilage. These findings clearly demonstrate that CSMA/PECA/GO hybrid scaffolds are suitable substrates for tissue engineering of articular cartilage. Futhermore, the electrical conductivity of the scaffolds may enhance the signal transduction between the cells and may be applied in other tissue engineering, such as nerve, bone *et al.*

## Methods

### Materials

Chondroitin sulfate A sodium salt (CS) (Type A 70%, balanced with Type C, from bovine trachea), glycidyl methacrylate (GMA), poly(ethylene glycol) methyl ether (MPEG, Mn = 2,000), ε-caprolactone (ε-CL), acryloyl chloride (AC), triethylamine (TEA) and ammonium persulfate (98%) (APS) were purchased from Sigma-Aldrich Company, USA. 2-(4-Amidinophenyl)-6-indolecarbamidine dihydrochloride (DAPI) was obtained from Roche, USA. The rest of the reagents were used as received. All the reagents were analytic grade quality.

### Macromer synthesis

#### Synthesis of methacrylated chondroitin sulfate (CSMA)

The synthesis of CSMA was followed the reference^17^,^52^. Briefly, CS was dissolved in 100 mL of phosphate-buffered saline (PBS, pH = 7.4), followed by addition of GMA (10 mL). The reaction solution was vigorously stirred at room temperature for 15 days. The macromer was then precipitated with acetone. The product was redissolved and extracted with chloroform to remove residual GMA. The water phase was concentrated, precipitated with acetone again, and dried under vacuum for 48 h. The CSMA was stored at −20 ^o^C.

#### Synthesis of poly(ethylene glycol) methyl ether-ε-caprolactone-acryloyl chloride (PECA) macromonomer

The MPEG-PCL copolymer was synthesized by ring-opening polymerization of ε-caprolactone initiated by MPEG using tin (II) 2-ethylhexanoate as catalyst, which was similar to the method reported before^53^,^54^. The obtained MPEG-PCL macromonomer dissolved in dewatered methylene chloride and reacted with AC at 40 ^o^C, refluxing for 4 h with a few drops of TEA to neutralize the formed HCl. Finally, the resultant PECA macromonomer was precipitated by petroleum ether and dried in vacuum at 25 ^o^C.

### Synthesis of graphene oxide (GO)

GO was synthesized by the reported method^55^ with some modification. In a typical synthesis procedure, a 9:1 mixture of concentrated 40 mL H_2_SO_4_/H_3_PO_4_ was added to a mixture of 0.3 g graphite flakes and 1.8 g KMnO_4_ with a slight exotherm (~30 min) to 35-40 °C. The reaction was then heated to 50 °C and stirred for 12 h. Thereafter, the reaction was cooled to room temperature and poured onto ice with 0.3 mL H_2_O_2_. For workup, the filtrate was centrifuged and the supernatant was decanted away. The remaining solid material was then washed in succession with 30 mL of water, 30 mL of concentrated HCl, 30 mL of anhydrous ethanol three times, and anhydrous diethyl ether. The obtained solid was vacuum-dried overnight at 35 °C temperature.

### Preparation and purification of CSMA/PECA/GO hybrid scaffold

With APS as the initiator agent, the CSMA/PECA/GO scaffold was synthesized by heat-initiated free radical method. The predetermined amount of PECA, CSMA, GO and APS were dissolved in water. From the whole dissolved components, equal amount of 200 μL was extracted and allocated sequentially to the holes in 96-well plate. Then, the entire system was soaked and bathed in water at 60 ^o^C for approximately 2 h. The obtained scaffolds were immersed in plenty of distill water for 2 h to remove any residual substances that did not react. Finally, the purified scaffolds were freeze-dried kept in air-tight bags before use. FTIR (KBr) spectra of the scaffold sample and the macromonomer were recorded on NICOLET 200SXV spectrophotometer (Nicolet). Scaffolds used in experments in vivo (both rats and rabbits) were sterilized by Cobalt-60 irradiation (Sichuan Academy of Agricultural Sciences, 25 kGy for 2 h).

### Characterizations of CSMA/PECA/GO hybrid scaffolds

The cross-section morphologies of the CSMA/PECA/GO scaffolds were observed by scanning electron microscopy (SEM, JEM-100CX, Japan), and the mean pore sizes were calculated from SEM images by JEM associated software. The porosity was measured by mercury intrusion porosimetry. For the swelling studies, dried scaffolds of each formulation were weighted (Wd)-prior immersion in pH = 7.4 PBS at 37 ^o^C. After 5 min, 10 min, 15 min, 30 min, 45 min, 60 min and 120 min of immersion, the samples were weighted (Ws) (n = 3). The superficial water was removed prior weighing with oil paper. The swelling ratio (Q) was obtained using the equation (Q = (Ws-Wd)/Wd). Meanwhile, the volume changes were also measured. The mechanical properties of the scaffolds with 1 cm radius and 0.5 cm high were determined by measuring their compression modulus in a wet state. An Instron 5500 mechanical tester with 10 KN load cell was used for compression mechanical test. The crosshead speed was set at 0.1 mm/min, and the load was applied until the scaffold was crushed completely. The conductivity of scaffolds prepared at different formulations was measured using a four-point probe measurement system with Keithley 220 programmable current source and Keithley 6514 programmable electrometer (Keithley Instruments Inc., USA).

### Cell cytotoxicity of CSMA/PECA/GO hybrid scaffold

The cytotoxicity of the CSMA/PECA/GO scaffold was analyzed by using 3T3 cells. The scaffold was extracted using DMEM for 24 h. Sequential dilutions of the stock solution were carried out to vary the concentrations of the leachates. The 3T3 cells were incubated with the leachates of different concentration for 1 day, 3 days and 5 days. At predetermined time, 20 μL of MTT was added to each well and the cells were further incubated for another 4 h. The precipitated formazan was dissolved in 160 μL DMSO and the absorbance at 570 nm was measured.

### Animals tests

For the animal experiment stage, twelve healthy Wistar rats weighting about 120 g were used to investigate in vivo biocompatibility of the scaffolds. They were purchased from Beijing HFK Bioscience Co., Ltd. Eighteen New Zealand White rabbits (initial weight 2-2.5 kg) were used. They were purchased from the Experimental Animals Center of Sichuan Province, China. The rabbits were evenly divided into three groups according to the pre-determined time points and fed with a standard laboratory diet. The animal care and use committee at Sichuan University approved all animal experiments conducted in this study. All methods were carried out in accordance with the approved guidelines (IACUC-S200904-P001).

### *In vivo* degradability and biocompatibility of CSMA/PECA/GO hybrid scaffold

*In vivo* biocompatibility of the CSMA/PECA/GO hybrid scaffold was investigated by implanting the materials into the subcutaneous of the back of Wista rats. At 1, 2, 4 and 8 weeks after surgery, three rats were sacrificed at each time point by using an overdose euthasate. The surgery sites were opened carefully, and then photograghs of the remaining scaffolds in rats were taken. The implants were harvested together with surrounding tissue for histological examination.

### Cell isolation and cell culture on CSMA/PECA/GO hybrid scaffold

The articular cartilages were removed from the knees joints of young New Zealand rabbits and cut into small pieces. Chondrocytes were released from cartilage slices by collagenase II (0.2 wt %) digestion. Following the complete dissolution of the matrix, the resultant cell suspension was filtered through a 40 μm Nitex filter and centrifugated at 2,000 rpm for 5 min. The isolated cells were then cultured in a Dulbecco’s Modified Eagle Medium (DMEM, Sigma) supplement with 10% fetal calf serum, 100 U/mL of penicillin and 100 μg/mL of streptomycin. They were incubated in an incubator at 37 ^o^C under 5% CO_2_, and the medium was changed every other day. As the cultured cells have grown to passage three, they were used to culture on scaffolds. The sterilized scaffolds were put into a 24-well culture plate and a total of 1.0 × 10^5^ cartilage cells were seeded on each well. The cell-seeded disk was maintained in the incubator for different time intervals (2, 4 and 7 days). The nucleus of the cells were stained with DAPI to take fluorescence photographs (DMIL, Leica, Germany). And the morphologies of the cells cultured on scaffolds were observed by SEM. The GAG content was assayed after reacting with dimethylmethylene blue dye and by measuring absorbance at 525 nm.

### Full-thickness cartilage defect repair

Full-thickness defect (thickness: 3 mm; diameter: 4 mm) was created through the articular cartilage and subchondral bone of the patellar groove in the left leg of the rabbits using an electric drill. The defects of rabbits were treated with CSMA/PECA/GO scaffolds injection with cartilage cells suspension (scaffolds + cells group, n = 9). Control defects included empty defects (n = 9) and defects filled with CSMA/PECA/GO scaffolds only (scaffolds group, n = 9). After intervention, the rabbits were housed under constant temperature (22 ^o^C) and were given tap water and food. Animals were kept in separate cages and were allowed to move freely. Samples were collected after 6, 12, 18 weeks and processed for further examination. All animal procedures were approved by the Sichuan University Committee on Animal Research and Ethics (IACUC-S200904-P001).

### Micro-computational tomography (Micro-CT)

The samples were analyzed using Micro-CT scanner (Y. Cheetah, YXLON International GmbH, Germany). The scan settings were: X-ray voltage = 55 kV, X-ray current = 90 μA, detector size = 1024 × 1024, and voxel resolution = 20 μm. The scans were then reconstructed to create 3D geometry using VGStudioMax. Three samples were analyzed at each time-point of harvest.

### Histological examination of repair tissue

For histological analysis, samples were fixed in 10% neutral buffered formalin and then embedded into paraffin. Sections were cut at 5 μm, deparaffinized and stained with Heamatoxylin and Eosin (H&E), Safrannin-O (Saf-O). All histological results were analyzed with a digital image analysis system (Nikon E600 Microscope with a Nikon Digital Camera DXM 1200, Nikon Corporation, Japan).

## Additional Information

**How to cite this article**: Liao, J.F. *et al.* Biodegradable CSMA/PECA/Graphene Porous Hybrid Scaffold for Cartilage Tissue Engineering. *Sci. Rep*. **5**, 9879; doi: 10.1038/srep09879 (2015).

## Figures and Tables

**Figure 1:**
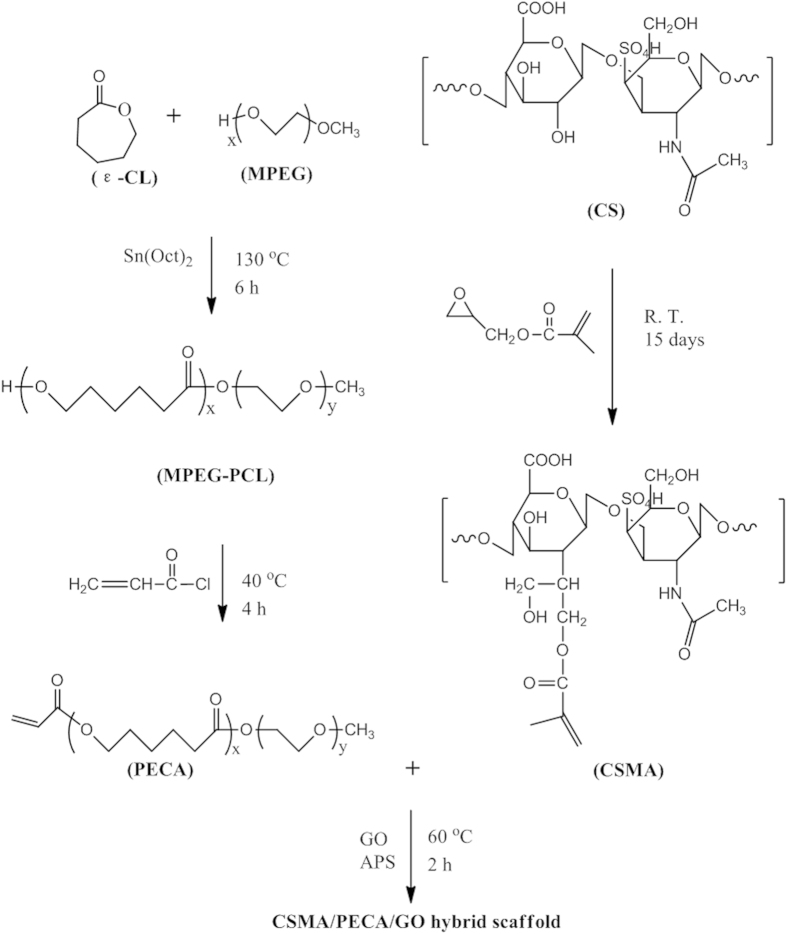
Synthesis of CSMA/PECA/GO hybrid scaffold.

**Figure 2:**
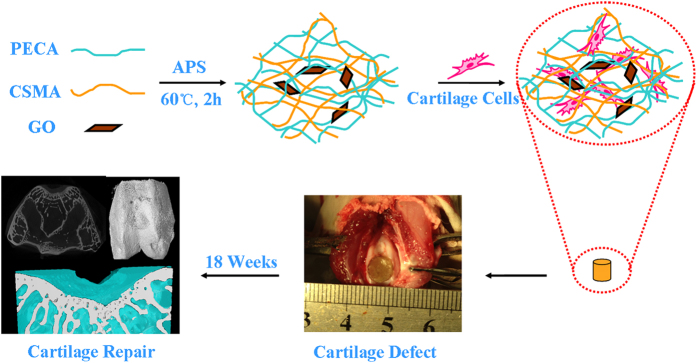
CSMA/PECA/GO hybrid scaffold for cartilage tissue engineering.

**Table 1:** Scaffolds preparation of different monomers content.

Code	CSMA:PECA	GO content (wt%)	APS Content (wt%)
**s-1**	5:1	3.0	22.2
**s-2**	4:2	3.0	22.2
**s-3**	3:3	3.0	22.2
